# Redefining development in *Streptomyces venezuelae*: integrating exploration into the classical sporulating life cycle

**DOI:** 10.1128/mbio.02424-23

**Published:** 2024-03-12

**Authors:** Evan M. F. Shepherdson, Marie A. Elliot

**Affiliations:** 1 Department of Biochemistry and Biomedical Sciences, McMaster University, Hamilton, Ontario, Canada; 2 Institute for Infectious Disease Research, McMaster University, Hamilton, Ontario, Canada; 3 Department of Biology, McMaster University, Hamilton, Ontario, Canada; Max-Planck-Institut fur terrestrische Mikrobiologie, Marburg, Germany

**Keywords:** *Streptomyces*, development, sporulation, siderophores, gene regulation

## Abstract

Two growth modes have been described for the filamentous *Streptomyces* bacteria. Their classic developmental life cycle culminates in the formation of dormant spores, where movement to new environments is mediated through spore dispersal. In contrast, exploratory growth proceeds as a rapidly expanding vegetative mycelium that leads to extensive surface colonization and is associated with the release of volatile compounds that promote alkalinization (and reduced iron bioavailability) of its surrounding environment. Here, we report that exploratory growth in *Streptomyces venezuelae* can proceed in tandem with classic sporulating development in response to specific nutritional cues. Sporulating exploration is not accompanied by a rise in environmental pH but has the same iron acquisition requirements as conventional exploration. We found that mutants that were defective in their ability to sporulate were unaffected in exploration, but mutants undergoing precocious sporulation were compromised in their exploratory growth and this appeared to be mediated through premature activation of the developmental regulator WhiI. Cell envelope integrity was also found to be critical for exploration, as mutations in the cell envelope stress-responsive extracytoplasmic function sigma factor SigE led to a failure to explore robustly under all exploration-promoting conditions. Finally, in expanding the known exploration-promoting conditions, we discovered that the model species *Streptomyces lividans* exhibited exploration capabilities, supporting the proposal that exploration is conserved across diverse streptomycetes.

**IMPORTANCE:**

*Streptomyces* bacteria have evolved diverse developmental and metabolic strategies to thrive in dynamic environmental niches. Here, we report the amalgamation of previously disparate developmental pathways, showing that colony expansion via exploration can proceed in tandem with colony sporulation. This developmental integration extends beyond phenotype to include shared genetic elements, with sporulation-specific repressors being required for successful exploration. Comparing this new exploration mode with previously identified strategies has revealed key differences (e.g., no need for environmental alkalinization), and simultaneously allowed us to define unifying requirements for *Streptomyces* exploration. The “reproductive exploration” phenomenon reported here represents a unique bet-hedging strategy, with the *Streptomyces* colony engaging in an aggressive colonization strategy while transporting a protected genetic repository.

## INTRODUCTION

*Streptomyces* are Gram-positive filamentous bacteria that are ubiquitous in the soil and the sediments of aquatic ecosystems. They are renowned for their specialized metabolism: they are a dominant source of natural products with antibiotic activity (1, 2), and produce a multitude of compounds with antifungal, immunosuppressant, pesticide, herbicide, and other bioactive properties (3–7). Beyond their impressive biosynthetic capabilities, *Streptomyces* have a complex multicellular, sporulating life cycle. This life cycle begins with spore germination, followed by hyphal tip extension and branching (8, 9). The resulting vegetative mycelium continues growing until reproductive growth initiates, where reproductive growth involves raising unbranched aerial hyphae and subsequently converting these hyphal filaments into chains of dormant spores. Recently, a new growth mode termed “exploration,” has been identified as an alternative to the classical sporulating developmental life cycle (10, 11). Exploration is characterized by the rapid outgrowth of vegetative-like hyphae across a solid surface, allowing these canonically non-motile bacteria to colonize areas far from their initial site of inoculation.

Multiple growth conditions are now known to induce exploratory growth, and exploration under these different conditions share several defining characteristics (10, 12). First, exploration is associated with a significantly increased rate of surface area expansion on solid medium, compared with growth during its classical life cycle. Second, exploring colonies develop unique surface morphologies characterized by networks of wrinkles, blisters, and buckles that are particularly prevalent near the colony center. And finally, exploring colonies emit trimethylamine, a small basic volatile organic compound that raises the pH of the surrounding area (10, 13). A major consequence of this environmental alkalinization is a corresponding decrease in iron bioavailability (13). This leads to growth inhibition of other microbes in the vicinity and serves as a chemical communication signal that both reinforces the exploration response in the producing *Streptomyces* colonies and promotes the initiation of exploratory behavior by nearby streptomycetes.

Iron limitation and adaptation to iron scarcity are critical for exploration success. *Streptomyces venezuelae* can secrete a suite of desferrioxamine siderophores that assist in surviving their self-imposed iron limiting environment (13). An additional siderophore (foroxymithine) is induced during growth on exploration plates supplemented with glycerol (12). Interestingly, while foroxymithine and desferrioxamine are partially redundant during monoculture growth, the two siderophores exhibit distinct spatial organization, and foroxymithine is critical for effective exploration in competitive culturing conditions (e.g., co-culture with yeast).

Here, we identify a new exploration-promoting condition that induces the growth of *S. venezuelae* in a way that combines characteristics of both exploratory and sporulating growth. This integration of disparate developmental pathways stemmed from the addition of glycerol to classical sporulation growth media. Transcriptional profiling revealed global changes in gene expression between exploration on this glycerol-supplemented medium relative to growth on its unsupplemented, classical development medium. In dissecting the regulation of these sporulating, exploring cells, we observed that the developmental regulators BldD and RsiG were required for wild-type growth, as was the extracytoplasmic function (ECF) sigma factor SigE. Unexpectedly, we discovered that exploration under these conditions was not accompanied by a rise in pH. Despite this lack of environmental alkalinization, iron availability and siderophore synthesis remained critical for growth and development, with the alternative foroxymithine siderophore being a key player. Finally, we show that glycerol supplementation of classical growth medium can not only promote the exploration of *S. venezuelae,* but it can also stimulate exploration in the model organism *Streptomyces lividans*, for which exploratory growth has not previously been observed.

## RESULTS

### Glycerol supplementation of sporulation-promoting medium stimulates exploratory-like growth

**Fig 1:**
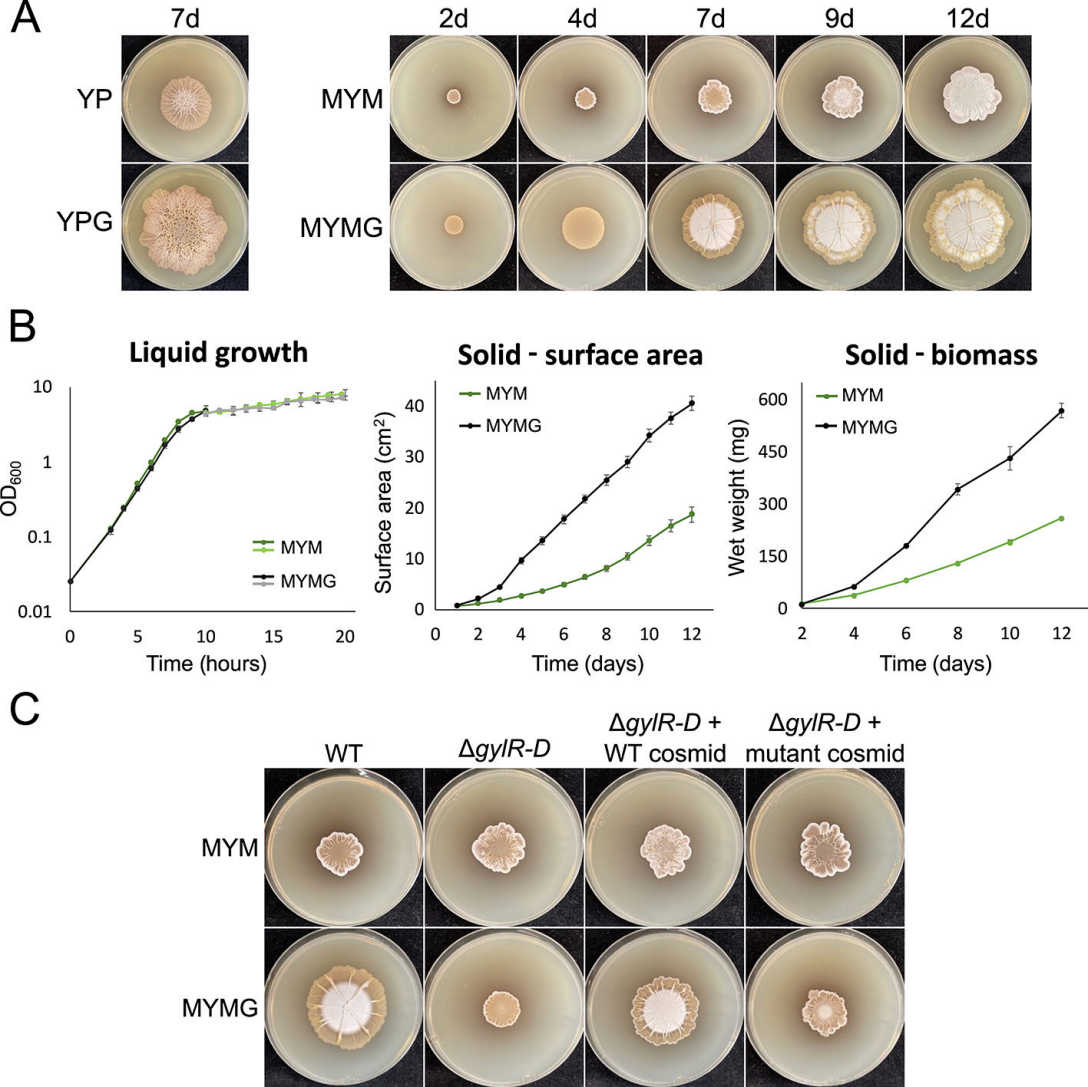
Growth of *S. venezuelae* on MYMG. (**A**) Left: photos of wild-type *S. venezuelae* exploring on YP and YPG (YP supplemented with glycerol) at 7 days of growth. Right: Photos of *S. venezuelae* spotted to MYM and MYMG over the course of 12 days. The same inoculum (volume and cell source) was used for each plate, and the plates were incubated at the same time. (**B**) Quantification of wild-type *S. venezuelae* growth rates in MYM and MYMG as measured by optical density at 600 nm (OD_600_) in liquid medium (left, three replicates per data point), colony surface area on solid medium (middle, six replicates per data point), and colony wet weight on solid medium (right, three replicates per data point). Error bars represent one standard deviation. Measurements for the liquid growth curve were performed for two sets of flask cultures, one over the first 10 hours of growth (darker lines) and one over the final 10 hours of growth (lighter lines). (**C**) Wild-type *S. venezuelae*, an isogenic glycerol catabolism operon mutant (Δ*gylR-D*), and Δ*gylR-D* complemented with wild-type copies of the genes on a cosmid or a vector control (mutant cosmid lacking the *gyl* genes) were spotted to MYM and MYMG and imaged after 7 days of growth.

Previous investigations into *Streptomyces* exploration revealed that the addition of glycerol to YP (yeast extract, peptone) medium dramatically enhanced the exploration phenotype (11, 12). Relative to unsupplemented plates, glycerol-grown colonies had an accelerated growth rate, colonized a larger surface area, and developed a more wrinkled surface morphology than previously reported exploring colonies (Fig. 1A). To gauge whether this glycerol response was confined to cells growing on YP-based media, we inoculated *S. venezuelae* strain NRRL B-65442 to a growth medium that supports classical sporulating development (malt extract, yeast extract, maltose, or MYM), both with and without added glycerol (MYM and MYMG, respectively). Unexpectedly, growth on MYMG shared characteristics with both classical development and exploration (Fig. 1A).

These MYMG-grown colonies formed radially extending wrinkles that were reminiscent of YP-grown explorer colonies (Fig. 1A) and displayed concentric rings of aerial hyphae and spores (where aerial growth appears as whitish regions on the plates), which had not previously been associated with exploration. Analogous to supplementing YP with glycerol (YPG), colonies on MYMG expanded significantly faster than colonies growing on MYM (Fig. 1A and B). Exploration-like colony growth/expansion thus appeared to be occurring concurrently with progression through the classical, sporulating life cycle in the central region of the colony (Video S1).

To further investigate the capacity of MYMG to support exploratory-like growth, we generated growth curves for *S. venezuelae* grown on solid or in liquid MYM and MYMG, and compared growth, surface area, and biomass accumulation (Fig. 1B). Glycerol supplementation of solid culture stimulated significant increases in both surface area and biomass relative to unsupplemented cultures, suggesting enhanced growth rates that were in line with colonies exploring on YP and YPG (11, 12). Surprisingly, these growth rate differences were confined to solid culture growth; in liquid, the growth profiles for MYM- versus MYMG-grown cultures were virtually indistinguishable (Fig. 1B). This suggested that exploration on solid culture may employ a different growth program than in liquid.

To eliminate the possibility that the MYMG phenotype on plates was mediated by altered physical properties of the growth medium as a result of glycerol supplementation, we inoculated wild-type *S. venezuelae* alongside an isogenic strain in which the operon responsible for glycerol uptake and catabolism was deleted. Growth of the mutant strain revealed a loss of rapid expansion capabilities on MYMG compared with wild type, and this phenotype could be complemented by reintroducing the glycerol uptake/catabolism operon (Fig. 1C). These results supported the use of an alternative growth strategy by wild-type *S. venezuelae*, where the rapid expansion of the wild-type strain required glycerol uptake and metabolism, and was not due to any biophysical properties conferred by glycerol.

We had previously seen that the enhanced exploration phenotype on YPG was specific to glycerol, with no other carbon source having an equivalent effect (12). To determine whether this was also true for MYMG, we tested the behavior of *S. venezuelae* growing on MYM supplemented with different carbon sources (Fig. S1). Many of the tested carbon sources (galactose, maltose, mannitol, sorbitol, and sucrose) gave rise to colonies that grew similarly to those without supplementation (MYM), with exceptions being arabinose (similar to glycerol/MYMG), succinate (faster expansion but with full complete sporulation), glucose (impaired aerial development and exploration), and acetate (no growth) (Fig. S1).

### Global transcriptional changes are observed between growth on MYM and MYMG

**Fig 2:**
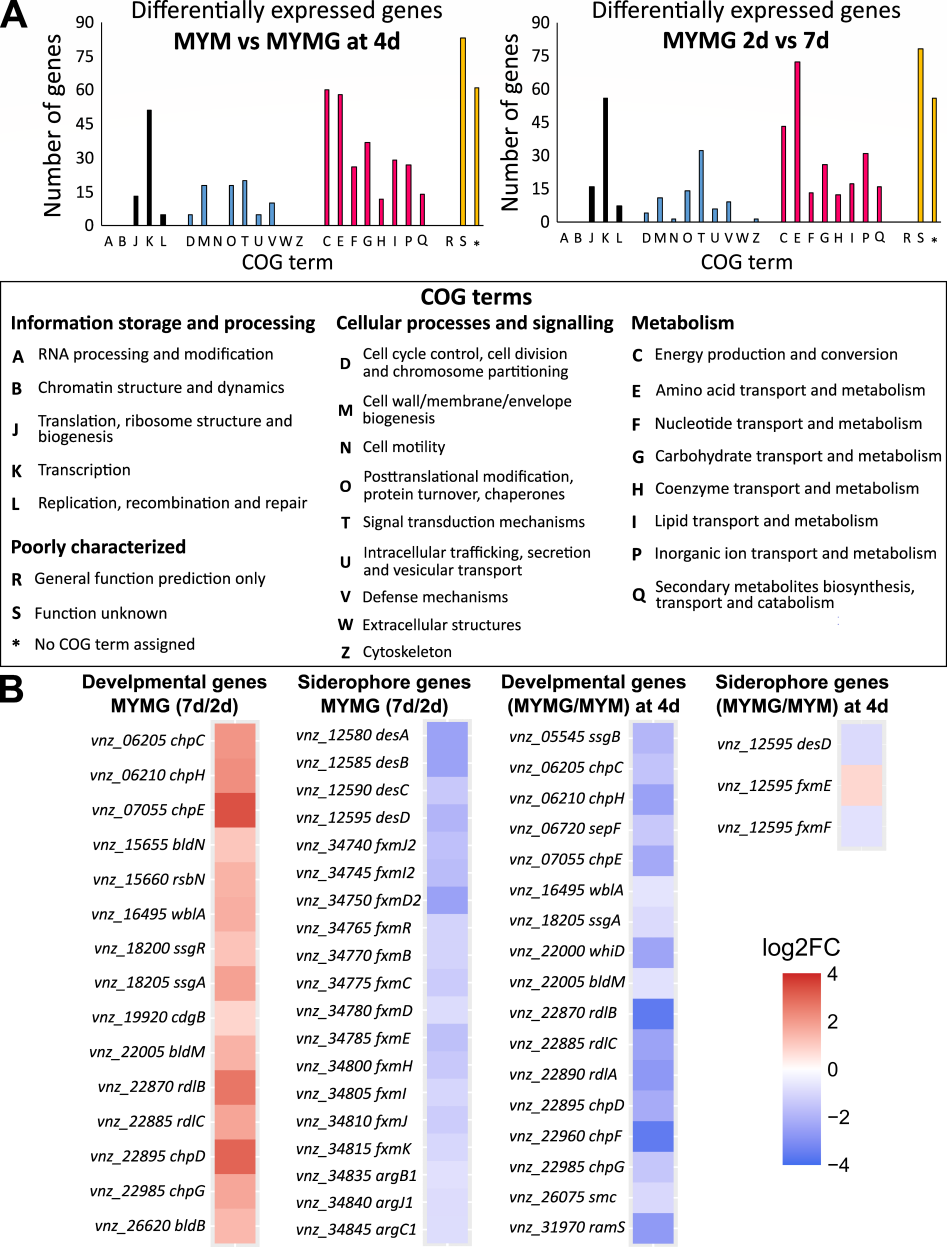
RNA-sequencing of MYM- and MYMG-grown colonies. (**A**) Differentially expressed genes from comparisons of samples isolated at 4 days from MYM- and MYMG-grown cultures, and MYMG at 2 days versus 7 days. These differentially expressed genes were sorted according to COGs and plotted according to the number of genes identified within each group, with the group designations indicated in the COG term list below. (**B**) Heat maps comparing significantly different (*P* < 0.05) transcript levels for classical developmental genes (first and third columns) and siderophore cluster genes (second and fourth columns) either over time on MYMG (first and second columns) or between MYM and MYMG (third and fourth columns). Blue, decreased expression; red, increased expression, as indicated by the log2 fold change (FC) scale on the right.

Given the distinct growth characteristics observed for MYMG- relative to MYM-grown cultures, and the similarities of MYMG-grown colonies to exploration, we sought to compare transcription profiles of colonies grown on MYM with or without glycerol supplementation. We isolated and sequenced RNA from colonies grown for 2 days (early), 4 days (mid), and 7 days (late) on MYMG, or 2 days and 4 days on MYM (e.g., Fig. 1A). Our transcriptomic data revealed global changes in gene expression, both between growth conditions and over time within a single condition. At 4 days, 521 genes were differentially expressed (adjusted *P*-value <0.05) when comparing MYM- and MYMG-grown cells, while 499 genes were differentially expressed when comparing early and late timepoints for MYMG. Functional categorization of the differentially expressed genes by Clusters of Orthologous Groups (COGs) revealed strong representation among genes associated with both primary and secondary metabolism (Fig. 2). Specifically, we found that many of the trends seen when comparing gene expression on YP and YPG (11, 12) were mirrored here. We observed changes in expression for genes involved in nitrogen metabolism, central carbon metabolism (glycolysis, citric acid cycle, and respiration), regulators of classical development (*bldM* and *wblA*) and classical development determinants (rodlin- and chaplin-encoding genes), as well as ECF sigma factors, and specialized metabolism/biosynthetic gene clusters, including those involved in the production of the antibiotic chloramphenicol and the siderophores desferrioxamine and foroxymithine (Tables S1 and S2).

### MYMG-mediated exploration is associated with a unique response to iron availability

We were intrigued by the higher expression of foroxymithine and desferrioxamine genes during the early stages of exploration on MYMG (Fig. 2B). A hallmark of *S. venezuelae* exploration in all previously identified growth conditions is the production of trimethylamine, which raises the pH of the surrounding medium and simultaneously reduces iron bioavailability. We found, however, that during growth on MYMG (and MYM), the medium pH remained neutral through 14 days of culturing. We had therefore expected that siderophore function may be less critical during exploration on MYMG than on other exploration-promoting growth media (where the rise in pH limited iron availability).

**Fig 3:**
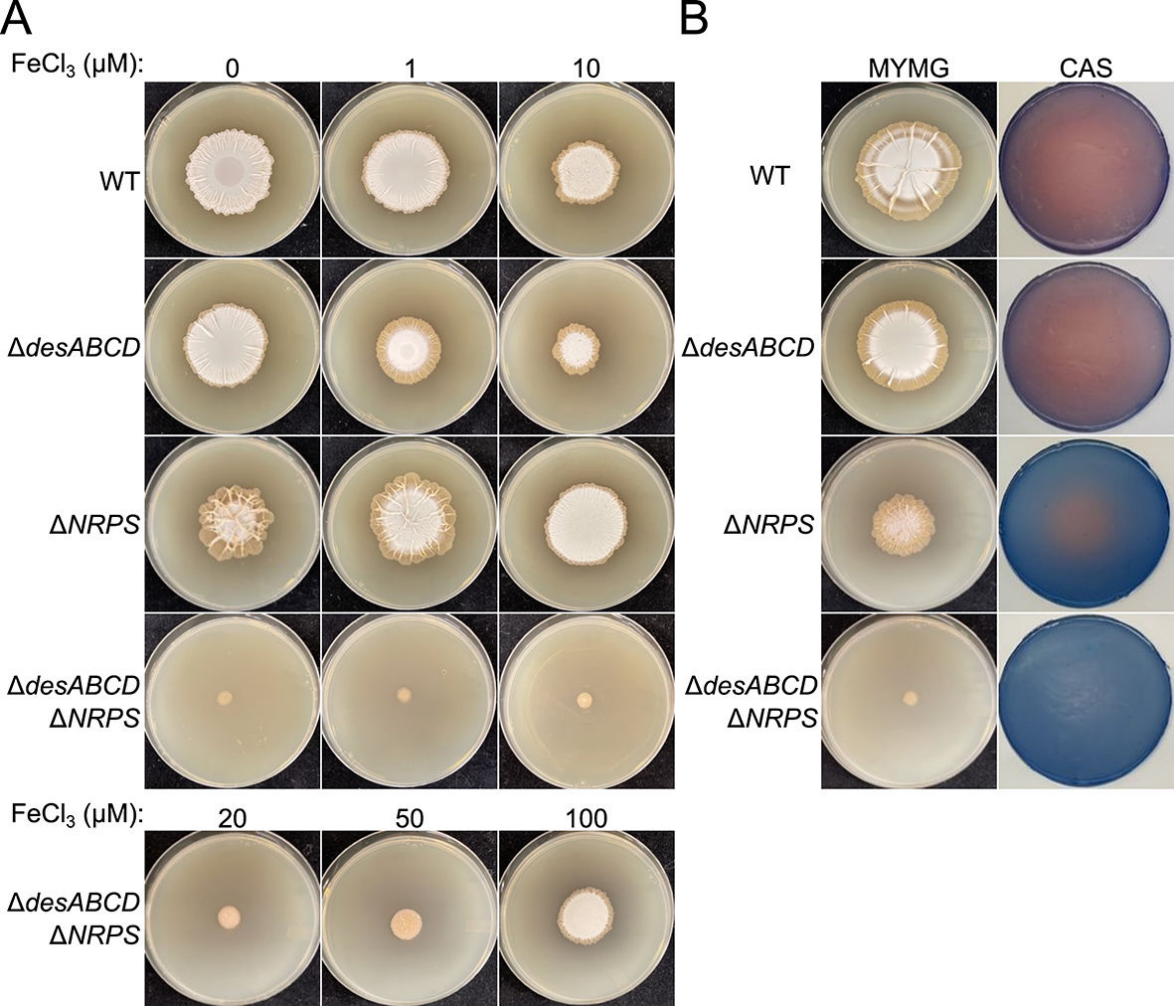
Effect of iron availability and siderophore repertoire on MYMG. (**A**) (Top) A panel of siderophore mutants defective in either desferrioxamine and/or foroxymithine production was spotted to MYMG supplemented with different concentrations of FeCl_3_. (Bottom) The mutant defective in both desferrioxamine and foroxymithine production was additionally spotted to MYMG supplemented with three higher concentrations of FeCl_3_. Images were taken after 7 days of growth. (**B**) The same panel of siderophore mutant strains were spotted to MYMG and imaged after 7 days of growth (first column). Chrome Azurol S (CAS) agar applied to the resulting conditioned medium was developed for 4 hours before being removed and imaged (second column), resulting in zones of color change local to where siderophores were active in the conditioned medium.

We had previously created individual and combined desferrioxamine and foroxymithine mutants (12, 13), and so we used these mutant strains to test the relative importance of these siderophores during growth on MYMG. We found that iron acquisition was important for exploration on MYMG: both siderophores made important contributions, but foroxymithine appeared to be the more important of the two. The desferrioxamine mutant exhibited wild-type growth, while the foroxymithine mutant grew slower and had reduced aerial development. In contrast, the double siderophore mutant did not raise aerial hyphae and failed to expand beyond the original site of inoculation (Fig. 3A, first/left column).

To evaluate whether the growth deficiencies associated with siderophore loss could be alleviated by iron supplementation, we inoculated the siderophore mutants onto MYMG supplemented with 1 or 10 µM FeCl_3_ (Fig. 3A, middle and right columns). We found that increasing concentrations of iron led to decreased colony expansion for both the wild-type and desferrioxamine mutant strains but restored wild type-like growth to the foroxymithine mutant at 10 µM (Fig. 3B). Interestingly, the double siderophore mutant displayed no growth improvement on MYMG supplemented with 10 µM iron, and so we tested the effects of further increasing iron levels (up to 100 µM) and found that partial phenotypic rescue required at least 50 µM iron supplementation (Fig. 3A, bottom row).

We knew from previous work on YPG-mediated exploration that desferrioxamine and foroxymithine exhibited differences in their spatial distribution, with foroxymithine diffusing beyond the colony borders, and desferrioxamine being confined to the colony area (12). We investigated their localization during *S. venezuelae* growth on MYMG using a Chrome Azurol S (CAS) colorimetric plate assay. CAS overlay plates revealed broad diffusion of siderophore activity in strains producing foroxymithine, whereas activity was limited to colony areas when desferrioxamine was the only siderophore produced (Fig. 3B). Collectively, these results suggested that increased diffusion of foroxymithine relative to desferrioxamine was a general (pH-independent) property of this siderophore, and that this siderophore was important for morphological development, and for colony exploration on multiple media types (YPG and MYMG).

### Regulators of classical development contribute to exploration on MYMG

**Fig 4:**
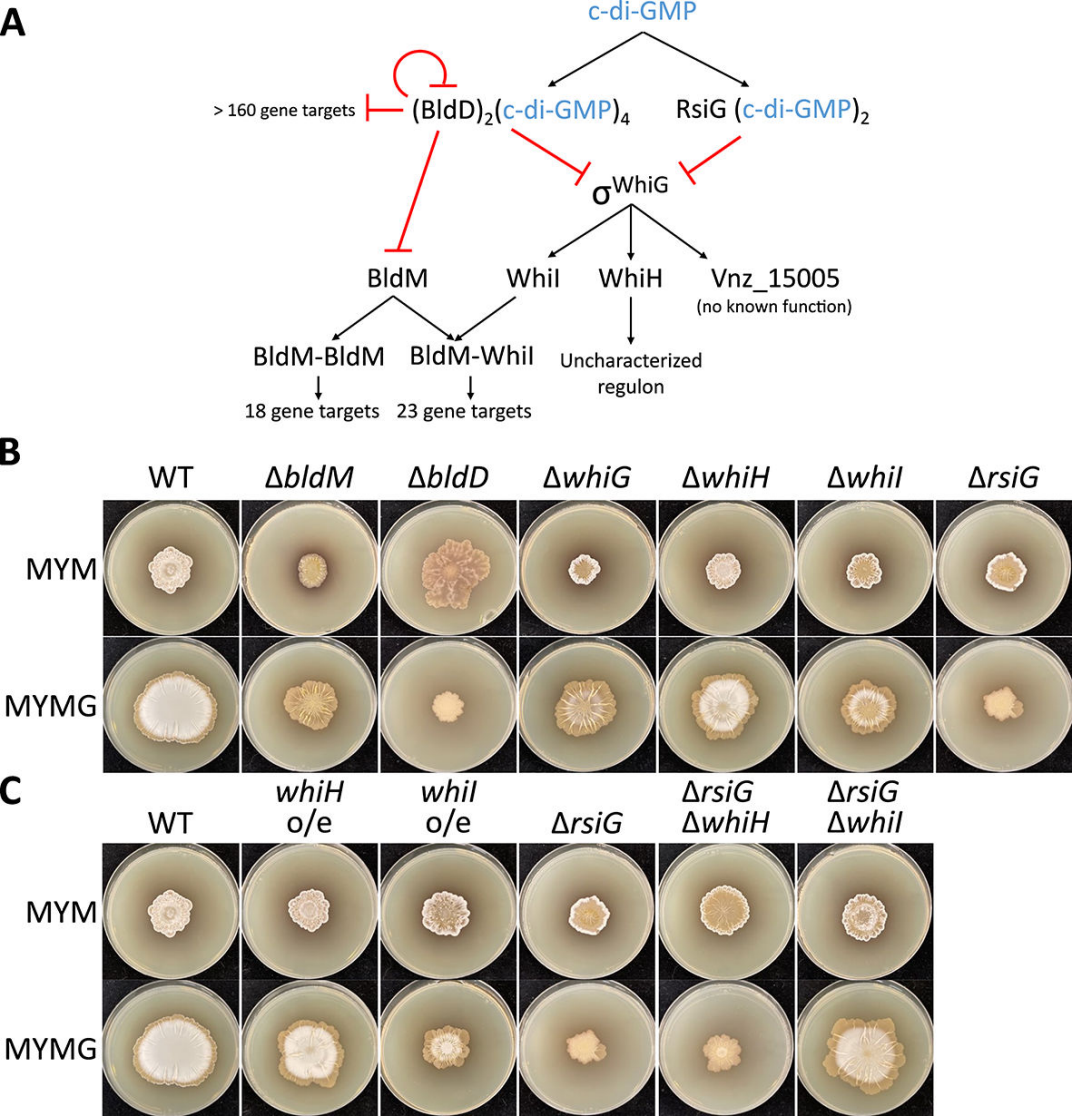
Regulators of classical development affect exploration on MYMG. (**A**) Abridged schematic of the regulatory cascade governing classical development, with a focus on the interplay between shared targets of BldD and WhiG. The BldD and RsiG co-factor cyclic-di-GMP (c-di-GMP) is indicated in blue, with its oligomeric state indicated in subscripted text. (**B**) Deletion strains for select regulators of sporulating development were spotted to MYM and MYMG and photographed after 7 days of growth. (**C**) The contribution of *whiH* and *whiI* to normal development across these same media types was further examined by spotting strains expressing each gene from a strong constitutive promoter (*whiH* o/e and *whiI* o/e) and by deleting each gene within a Δ*rsiG* mutant background. Images were taken after 7 days of growth (wild-type and *rsiG* mutant images are duplicated in panels B and C to facilitate comparisons between strains).

Beyond the dynamic expression of siderophores during growth on MYMG medium, we also observed differential expression for many genes whose products promote aerial hyphae formation and alter the surface properties of the aerial hyphae and spores (e.g., chaplin, rodlin, and SapB-encoding genes) (Fig. 2B; Tables S1 and S2). Notably, these genes are direct and indirect targets for many classical developmental regulators (Fig. 4A). Given this, and given the interesting sporulation characteristics of the wild-type strain during exploration on MYMG, we wanted to determine whether development and exploration were co-regulated under these growth conditions, as no strong connections had been observed previously (10, 14). We inoculated a collection of classic developmental mutants (predominantly *bld* and *whi* mutant strains that are defective in aerial hyphae formation or sporulation, respectively; Fig. 4A) to MYM and MYMG, to assess whether these gene deletions perturbed growth under distinct sporulation- and exploration-promoting conditions (Fig. 4B).

On MYM, as expected, the *bld* mutants failed to raise aerial hyphae (although the *bldD* mutant colony exhibited a colony morphology that was reminiscent of exploration on YP), while the *whi* mutants failed to sporulate. In contrast, on MYMG, the growth of these mutants partitioned into two categories. Mutations that blocked development (Δ*bldM*, Δ*whiG*, Δ*whiH*, and Δ*whiI*) did not prevent exploratory growth on MYMG, with colonies exhibiting expansion and wrinkling patterns that were reminiscent of wild-type exploration on MYM and YP media, although they differed from wild type and each other in their surface characteristics. In contrast, mutations that accelerated development and led to hypersporulation (Δ*bldD* and Δ*rsiG*) abrogated exploration on MYMG.

To gain insight into the mechanism underlying this exploration repression, we focused our attention initially on RsiG, as its downstream regulatory network is much less complex than that of BldD (Fig. 4A). RsiG is the cognate anti-sigma factor of the sporulation sigma factor WhiG (15, 16). WhiG directs the transcription of three genes in *S. venezuelae*: the developmental regulator-encoding genes *whiH* and *whiI*, and a poorly characterized hypothetical protein-encoding gene *vnz_15005* (15). Thus, in the *rsiG* mutant, we expected precocious WhiG activity and premature/hyper-expression of its target genes.

To determine whether a WhiG target gene(s) was responsible for the *rsiG* phenotype, we independently cloned *whiH* and *whiI* downstream of a strong constitutive promoter (ermE*). The resulting constructs were introduced into a wild-type background to test whether either could recapitulate the Δ*rsiG* phenotype (Fig. 4C). In parallel, we created *whiH* and *whiI* deletions in the Δ*rsiG* background to test whether eliminating the downstream targets of RsiG could rescue exploratory growth on MYMG (Fig. 4C). We found that constitutive, high-level *whiH* expression in a wild-type background had little effect on the exploration phenotype on any medium type, while equivalent expression of *whiI* in the same background impaired growth on MYMG. Consistent with these observations, deleting *whiH* in the Δ*rsiG* background had no impact on the *rsiG* mutant phenotype on MYMG, whereas a Δ*rsiG* Δ*whiI* double mutation rescued the *rsiG* exploration defect (Fig. 4B). Together, these data strongly implicated early overexpression of *whiI* as being detrimental to exploratory growth on MYMG.

WhiI associates with BldM to form a BldM-WhiI heterodimer that activates the expression of late-sporulation specific genes (17) (BldM can also form a homodimer that controls an independent set of genes; WhiI does not appear to function on its own). Given this, we wondered whether overexpressing *bldM* would have the same effect as overexpressing *whiI*. We found that unlike the situation with *whiI,* overexpressing *bldM* had no significant effect on exploration (Fig. S2). This suggested that either premature assembly of the BldM-WhiI heterodimer adversely affects exploration, or contrary to what has been observed during classical development (17), WhiI may act independently of BldM during exploration. We took advantage of previous chromatin immunoprecipitation (ChIP) sequencing analyses of BldM and WhiI, and identified one target gene (*murA2*) that appeared to be bound more strongly by WhiI than BldM based on ChIP enrichment scores, making it a plausible candidate for WhiI-specific control (17). However, we found that overexpressing *murA2* in a wild-type background did not recapitulate the *whiI* overexpression phenotype (Fig. S2). This suggested that it was not responsible for the effect observed, and that the exploration defect may be the result of premature BldM-WhiI assembly, although we cannot formally exclude the possibility that during exploration, WhiI acts alone to control an independent set of genes.

### The cell envelope-responsive sigma factor SigE is required for exploration under diverse growth conditions

**Fig 5:**
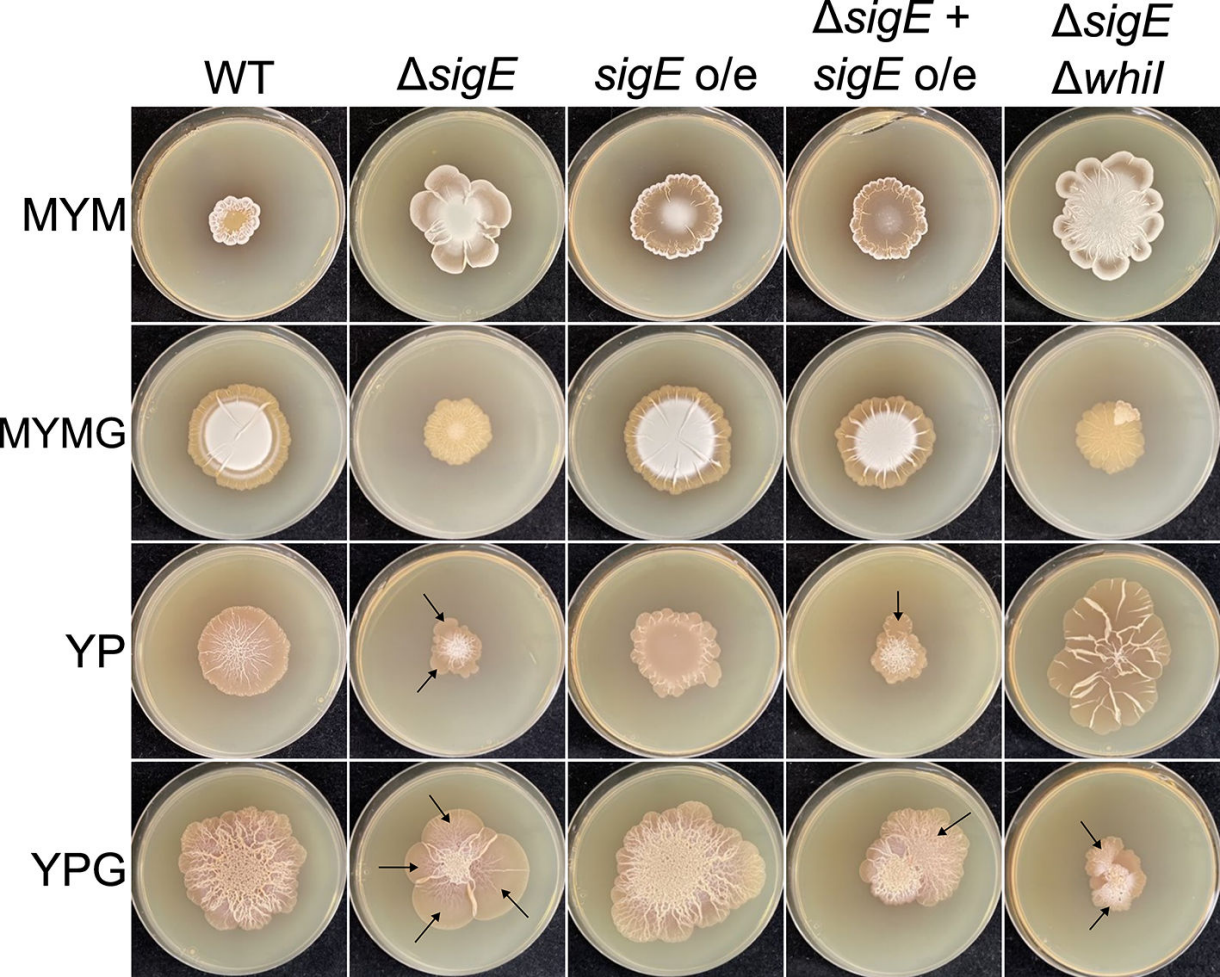
SigE function is important for robust exploration. Strains of *S. venezuelae* in which the cell envelope stress-responsive extracytoplasmic sigma factor *sigE* was deleted or placed under a strong constitutive promoter (*sigE* o/e) were spotted to different media. A complemented strain (where *sigE* was cloned under the control of the strong constitutive ermE* promoter and introduced into the deletion mutant; Δ*sigE + sigE* o/e) and a combined double-deletion mutant for *sigE* and *whiI* were also spotted to the same media. Images were taken after 7 days of growth. Emerging suppressor mutants are indicated with black arrows.

In addition to a role for developmental regulators in controlling sporulation-associated exploration, we also identified multiple ECF sigma factors whose genes were differentially expressed (both over time for MYMG-grown cultures, and between MYM and MYMG cultures), including the broadly conserved *sigQ* (Tables S1 and S2). *sigQ* had previously been identified as a differentially expressed gene in earlier exploration (YP/YPG) RNA-sequencing data sets (11). We tested the exploration capabilities of *sigQ* mutant and overexpression strains on MYMG, and observed only subtle differences relative to wild type during growth on either MYM or MYMG (Fig. S3). The ECF sigma factor *sigE* had also emerged as a target of interest in previous analyses (11, 12). In *Streptomyces*, SigE is responsible for maintaining cell envelope integrity, and its activation upon sensing envelope stress by the two-component system CseBC leads to the upregulation of a large regulon in *Streptomyces coelicolor*, many of which encode cell wall- or cell envelope-associated enzymes (18–21). As the rapid growth rate associated with exploration may require greater investment to maintain cell envelope integrity relative to slower growing classically developing colonies, we tested the effects of overexpressing and deleting *sigE* under a range of exploration growth conditions. In contrast to *sigQ*, multiple independently generated *sigE* deletion mutant strains were found to be significantly impaired in exploration on YP, YPG, and MYMG (Fig. 5), but grew well on the classical development medium MYM. Overexpressing *sigE* had minimal effects. Curiously, introduction of the overexpression construct into any of the Δ*sigE* mutants could fully complement growth of the mutant on MYMG but not on YP or YPG.

On YP and YPG, but not MYMG, the Δ*sigE* mutant consistently acquired suppressor mutations that resulted in irregular, rapidly exploring outgrowths extending from the main body of the colony (Fig. 5). As our investigation into the effects of classical developmental regulators revealed that inappropriate signaling downstream of WhiI negatively impacted exploration on MYMG, we tested whether deleting *whiI* in the Δ*sigE* background could rescue the MYMG phenotype as it had for Δ*rsiG*. We found that while the Δ*sigE* Δ*whiI* mutant was effectively indistinguishable from the *sigE* mutant when grown on MYMG and YPG, exploration was rescued—and even enhanced albeit with very different colony architecture—during growth on YP (Fig. 5; final column). These observations collectively suggested that exploration is profoundly impacted by SigE, and that there may be distinct downstream regulatory programs that are activated depending on the growth conditions.

### MYMG can promote exploration in species that do not explore under conventional exploration conditions

**Fig 6:**
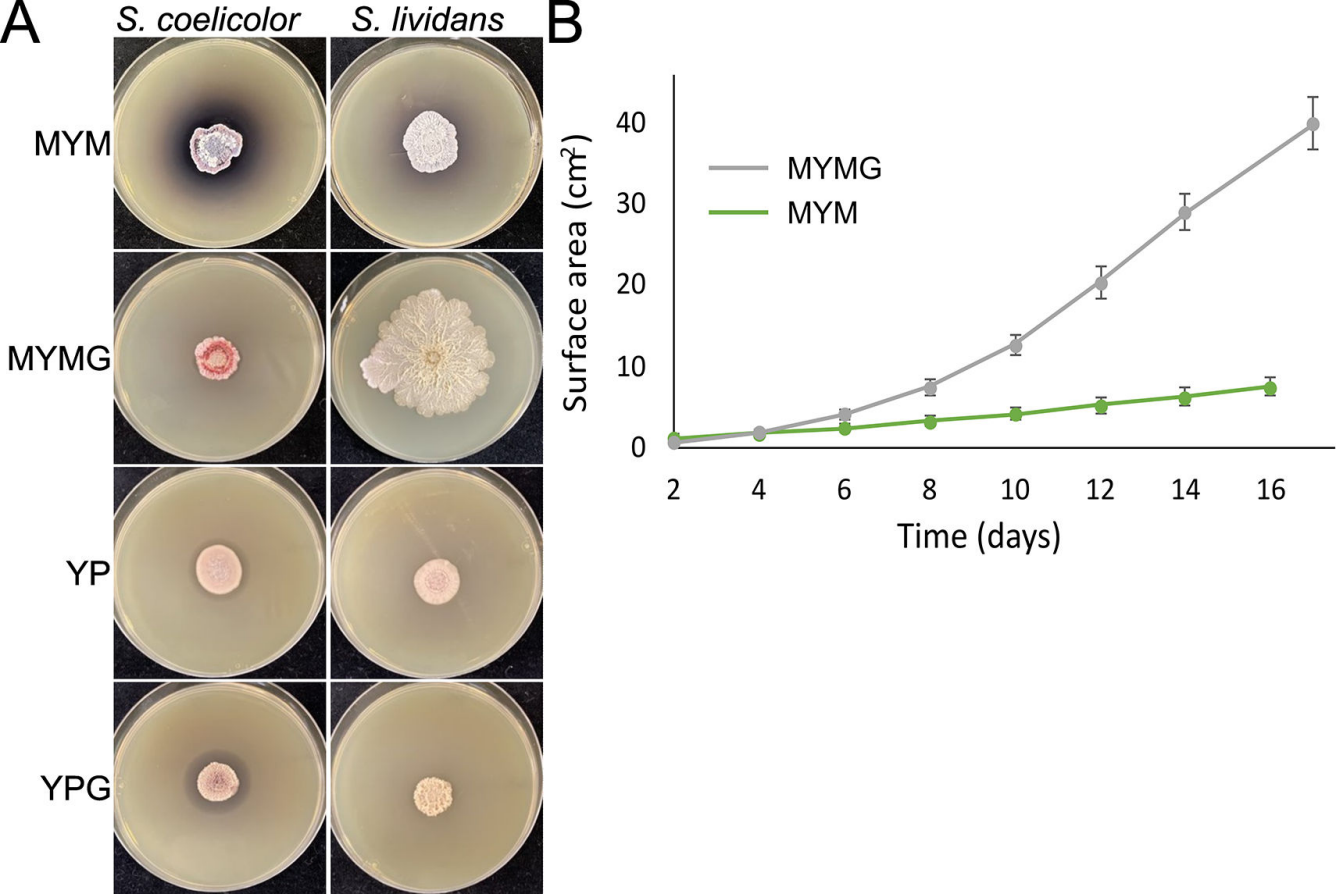
*Streptomyces lividans* displays exploratory growth on MYMG. (**A**) Growth of the model species *S. coelicolor* and *S. lividans* on MYM, MYMG, YP, and YPG. All photos were taken after 14 days of growth. (**B**) Graph of *S. lividans* surface area over time, during growth on MYM and MYMG.

When exploration was first described, it was reported that ~10% of wild *Streptomyces* isolates (out of 200 tested) could explore when grown in co-culture with yeast (10). A key question has been whether exploration capabilities are confined to a subset of streptomycetes, or if it is widespread but the initiating stimuli had yet to be identified. We set out to investigate whether our increasingly diverse panel of exploration-promoting growth conditions could stimulate exploratory growth in model *Streptomyces* species not known to explore. We focused our attention on *S. coelicolor* and *S. lividans*, and inoculated them on MYM, MYMG, YP, and YPG (Fig. 6A). Consistent with previous studies, after 14 days of growth, no exploration was observed for either strain on YP or YPG, and robust sporulation was observed for both on MYM. On MYMG, however, *S. lividans* appeared to be exploring: the colony expanded to cover a much larger area of the plate than when grown on MYM (Fig. 6B), and it developed a complex network of wrinkles with a highly structured core (Fig. 6A; Video S2).

We knew that *sigE* was required for *S. venezuelae* exploration on MYMG. To determine whether the same genetic requirements were conserved in *S. lividans*, we deleted the ortholog of *sigE*. Consistent with our *S. venezuelae* observations*,* loss of *sigE* in *S. lividans* resulted in wild-type growth on MYM but severely impacted exploration on MYMG (Fig. S4).

## DISCUSSION

*Streptomyces* exploratory growth has historically been viewed as proceeding independently of its classical sporulating life cycle (10, 14). Our findings here reveal that exploration can in fact be effectively integrated into the conventional reproductive growth cycle in a nutrient-dependent manner, with glycerol supplementation (or supplementation with arabinose or succinate) promoting both sporulation and exploration. We focused our attention here on glycerol supplementation, given our previous work in this area; however, the ability of specific carbon sources—and not others—to enhance exploration suggests a need for greater understanding of carbon metabolism and nutrient flux in these bacteria.

The discovery that exploratory growth could also proceed without growth medium alkalinization led us to revisit the characteristics that define exploratory growth. When comparing exploration on MYMG to exploration on other media types, three universally shared characteristics were identified. First, exploration involves rapid colony expansion that appears to be driven by enhanced growth, coupled with possible surfactant-mediated sliding along the solid growth substrate. Second, exploring colonies adopt a highly wrinkled colony architecture reminiscent of biofilms formed by other bacteria. And finally, exploration requires the function of the sigma factor SigE, which in *Streptomyces* bacteria is responsible for initiating a cell wall stress response (18, 19, 21). There also appear to be common environmental or nutritional factors that impact exploration, including glucose levels (abundant glucose represses exploration), glycerol levels (abundant glycerol enhances exploration), and iron levels (multiple iron sequestration strategies are employed by exploring cultures).

Previous work has revealed that trimethylamine production and release by exploring colonies is a powerful mediator of change in the dynamics and composition of the surrounding microbial community (10, 13). In particular, the resulting environmental alkalinization promotes exploratory behavior in other streptomycetes, and simultaneously inhibits the growth of other microbes by reducing bioavailable iron. The discovery here of an exploration mode that does not involve medium alkalinization suggests there may be situations where such widespread environmental modulation is not advantageous. This “stealth” mode of sporulating/reproductive exploration was not observed under conditions of self-induced environmental alkalinization, suggesting that these cells may be responsive to different community pressures. We did note, however, that MYMG-grown exploring colonies retained the ability to effectively complete for iron with other microbes on a more local scale through their release of the diffusible foroxymithine siderophore. Foroxymithine can function to reserve iron for its producer strain, and consequently, these exploring colonies would be expected to influence iron availability in their immediate vicinity. A future priority will be to probe these different exploration behaviors to better understand their associated fitness costs and benefits.

How exploration is genetically regulated remains an important question across all stimulating media conditions. While exploration by developmental mutants on YP and YPG can proceed at varying rates, exploration on MYMG was arrested in Δ*bldD* and Δ*rsiG* strains, likely due to inappropriately high levels of WhiI activity [*whiI* expression is indirectly controlled by both RsiG and BldD (15, 22, 23)]. The role of BldD and RsiG as important determinants of MYMG exploration success is particularly interesting as these are two of the three known cyclic-di-GMP (c-di-GMP)-binding protein effectors in *Streptomyces* (the third being the glycogen debranching enzyme GlgX, which impacts energy storage) (15, 16, 24, 25). Nucleotide second messengers like c-di-GMP have widely been reported to influence population-level behaviors like motility and biofilm formation in many bacteria (26–30). Whether nucleotide second messengers contribute to the initiation or continuation of exploratory growth is an area of interest for future studies.

While the requirements of functional BldD and RsiG were unique to MYMG-mediated exploration, the requirement of SigE for successful exploration was consistent across multiple conditions (MYMG, YP, and YPG), and different *Streptomyces* species. Indeed, the observation of exploratory growth by *S. lividans* and the conservation of genetic controls across the phylogenetically diverse *S. venezuelae* and *S. lividans* suggest that exploratory growth is a broadly conserved developmental adaptation in the streptomycetes, and is one that can be triggered by distinct environmental conditions. In the case of *sigE,* its expression is induced in response to cell envelope stress (18–20). These findings suggest that irrespective of the growth condition, entry into exploration may be accompanied by perturbation of the cell wall or membrane, and that the upregulation of σ^E^-target genes [e.g., those encoding cell wall biosynthetic enzymes; (18, 19, 21)] is needed to resolve this stress. Analogous connections between cell wall integrity and biofilm formation have been reported previously for other microbes, including *Bacillus subtilis* (31), *Staphylococcus aureus* (32), and *Aspergillus fumigatus* (33).

The discovery that *Streptomyces* sporulation and exploration are not mutually exclusive provides interesting bet-hedging opportunities. Exploring colonies move rapidly along solid surfaces and enable colonization of areas distant from their site of inoculation. In contrast, sporulation provides a reservoir of dormant, metabolically inactive cells that can resist many environmental insults. Sporulation by exploring cultures could therefore provide the mobile exploring population with a protected genetic reservoir that would be impervious to nutritional challenges or attack by phage or other microbes, ensuring colony survival as the exploring cells enter new territories. At the same time, the exploring colony offers a novel mechanism of spore dispersal, either directly transferring spores to new environments, or moving them to locations where they may be better dispersed by other factors (e.g., wind, insects).

In other sporulating microbes like *Bacillus subtilis,* the decision to sporulate, form a biofilm, or migrate is not mutually exclusive at a population level, and is increasingly being recognized as outcomes that exist along a regulatory continuum (34, 35), with individual cells or subpopulations having defined—and distinct—roles within the larger population (36). Our results are suggesting that there are shared regulatory elements that contribute to both exploration and sporulation in the streptomycetes, and understanding the interplay between vegetative growth, cell dormancy, biofilm formation, and mobility will be an important goal for the future.

## MATERIALS AND METHODS

### Strains, plasmids, media, and culture conditions

Strains, plasmids, and primers used in this study are listed in Tables S3 to S5, respectively.

*S. venezuelae* NRRL B-65442 was grown in liquid MYM (1% malt extract, 0.4% yeast extract, 0.4% maltose) for overnight cultivation and on solid MYM (2% agar) for spore stock generation. Spore stocks for wild isolates were similarly prepared. Spore stocks for *Streptomyces coelicolor* M145 and *Streptomyces lividans* 1326 were prepared from lawns cultivated on cellophane discs on top of solid MS (2% soya flour, 2% mannitol, 2% agar). When studying growth on solid media, 10 µL of an overnight culture of *S. venezuelae* was spotted onto 40 mL plates containing 2% agar plus additional nutrients [YP (1% yeast extract, 2% peptone), YPG (YP with 2% glycerol), MYM, or MYMG (MYM with 2% glycerol)]. For both solid and liquid media, YP and YPG were prepared using deionized water, while MYM and MYMG were prepared using tap water. Solid MYM or MYMG plates were additionally supplemented with 2% carbon source (e.g., arabinose or galactose) or iron (III) chloride (1 µM, 10 µM, 20 µM, 50 µM, or 100 µM) where appropriate. All *Streptomyces* cultures were grown at 30°C.

### Construction of *Streptomyces* mutant strains

Gene deletions were generated using ReDirect technology (37). Coding sequences on a cosmid vector carrying large fragments (30 to 40 kb) of genomic DNA from *S. venezuelae* or *S. coelicolor* were replaced by an *oriT*-containing apramycin (Δ*whiH*/Δ*vnz*_*27205*; Δ*whiI*/Δ*vnz*_*28820*; Δ*sigE/*Δ*sli*_3698 in *S. lividans*, using *S. coelicolor* cosmid StE94) or hygromycin (Δ*whiI*/Δ*vnz*_*28820* or Δ*whiG*/Δ*vnz_26215*) resistance cassette. In creating the *S. venezuelae sigE*/*vnz_15840* deletion strain, the coding sequence and flanking 4–5 kb upstream and downstream sequences were PCR amplified and cloned into the pCR2.1-TOPO vector between the HindIII-SpeI and SpeI-XbaI restriction sites (this region lacked an appropriate cosmid for use in creating the gene deletion) before the *sigE* gene was targeted for replacement with the apramycin resistance cassette as described above. The *rsiG* deletion construct was generated through Gibson assembly of four DNA fragments generated by PCR (the vector backbone of pCR2.1-TOPO, a 4.5 kb fragment of *S. venezuelae* genomic DNA upstream of *rsiG*, an *oriT*-containing hygromycin resistance cassette, and a 4.7 kb fragment of *S. venezuelae* genomic DNA downstream of *rsiG*). The mutant cosmids/plasmids were introduced into the non-methylating *Escherichia coli* strain ET12567/pUZ8002, followed by conjugation into *S. venezuelae*. The resulting exconjugants were screened for double-crossover events, and gene deletions were verified by PCR using combinations of primers located upstream, downstream, and internal to the deleted regions (Table S5).

To generate the *bldM, whiH,* and *whiI* overexpression constructs, a 289 bp fragment containing the ermE* promoter was amplified from pIJ12251 with primers incorporating AvrII and HindIII restriction enzyme recognition sites (Table S5) and the resulting amplicon was then cloned into pMS82 following digestion with AvrII and HindIII. Coding sequences for *bldM*/*vnz_22005, whiH*/*vnz_27205,* and *whiI/vnz_28820* were subsequently amplified from *S. venezuelae* genomic DNA, *S. venezuelae* cosmid 4O01, or *S. venezuelae* cosmid Sv-6-D05, respectively, with primers that contained 5′ HindIII and KpnI restriction enzyme recognition sites (Table S5). The resulting amplicons were then cloned into the pMS82-ermE*p construct following digestion with HindIII and KpnI (Table S4).

To generate the *sigE* and *murA2* overexpression constructs, a 487 bp fragment containing the ermE* promoter was digested out from pIJ12251 using PvuII and EcoRV and subcloned into EcoRV-linearized pMS82. The coding sequence of *sigE* (*vnz_15840*) was amplified from *S. venezuelae* genomic DNA with primers incorporating 5′ NdeI and XhoI restriction enzyme recognition sites and *murA2* (*vnz_28735*) was amplified from *S. venezuelae* genomic DNA with primers incorporating 5′ NdeI and SpeI restriction enzyme recognition sites (Table S5). The resulting amplicons were then cloned into the pMS82-ermE*p construct following digestion by NdeI and XhoI or NdeI and SpeI (Table S4).

### Time-lapse videos of exploring colonies

Ten microliters of overnight *S. venezuelae* or *S. lividans* cultures was spotted onto solid medium, and the plates were then placed on an Epson Perfection V800 photo scanner in a 30°C incubator. Images were acquired every hour, and the resulting time course images were compiled in a video format as sequential single frames.

### Growth curves in liquid medium

Wild-type *S. venezuelae* grown overnight in 10 mL MYM was used to inoculate 50 mL of fresh MYM or MYMG in baffled flasks to an initial optical density at 600 nm (OD_600_) of 0.025. OD_600_ measurements were taken every hour from 3 to 10 hours of growth for one batch of flasks and from 10 to 20 hours for a second batch of flasks. Technical triplicates were performed for each growth condition from a single biological replicate.

### Growth curves in solid medium

Photos of growing colonies were taken at set time intervals. Surface area analyses were performed using ImageJ. The scale was established for each image by setting the diameter of the Petri dish to 10 cm. The perimeter of the colony was traced, and the area of the captured region was determined. Six replicates were analyzed for each timepoint and condition. For determining changes in colony biomass over time, a single wild-type *S. venezuelae* overnight culture (10 mL MYM) was used to inoculate (10 µL) MYM and MYMG plates. Every 2 days from 2 to 12 days post-inoculation, whole colonies were scraped from the medium and their wet weight determined using an analytical balance. Three colonies were analyzed for each timepoint and condition.

### RNA isolation, library preparation, and cDNA sequencing

RNA was isolated, as described previously (38), from two independent replicates of *S. venezuelae* grown on solid medium for 2, 4, or 7 days. rRNA was depleted from all samples using a Ribo-Zero rRNA depletion kit. cDNA and Illumina library preparation were achieved using a NEBNext Ultra directional library kit, followed by sequencing using un-paired-end 80 bp reads on the MiSeq Illumina platform. Bioinformatic analyses were conducted using the free open-access platform Galaxy (https://usegalaxy.org). Reads were aligned to the *S. venezuelae* genome using Bowtie2 (39); after which, they were sorted, indexed, and converted to BAM format using SAMtools (40). Transcript level normalization and differential transcript level analyses were conducted using DESeq2 (41). DESeq2 transcript normalization and differential gene expression between MYM and MYMG at 4 days of growth involved comparing transcripts sequenced from those specific timepoints/conditions as input data; DESeq2 transcript normalization and differential gene expression between MYMG at 2 days and 7 days of growth in turn involved comparing transcripts sequenced from those specific conditions as well as MYMG at 4 days of growth as input data. COG analysis was performed using EggNOG-mapper (42). RNA-sequencing data have been submitted to the National Center for Biotechnology Information Gene Expression Omnibus (NCBI-GEO) repository and assigned the accession number GSE240807.

### Chrome Azurol S assay for siderophore activity

Samples containing siderophores of interest were assessed using a Chrome Azurol S-based assay as described previously (12). Briefly, sterile solid medium containing the colorimetric dye (2% agar, 100 µM Chrome Azurol S, 200 µM hexadecyltrimethylammonium bromide, 10 µM FeCl_3_, 0.5 M 2-(N-morpholino)ethanesulfonic acid, pH 5.5) was poured as 15 mL plates. To measure siderophore activity for conditioned medium *in situ*, 15 mL of CAS agar was prepared and poured as described above into a 10 cm Petri dish. After 7 days of growth, biomass was removed from the surface of the conditioned solid medium, followed by overlaying of the conditioned medium with CAS agar. These sandwiched agar plates were then incubated in the dark at room temperature for 4 hours; after which, the CAS agar layer was removed and photographed.

## Data Availability

RNA-seqsequencing data hasve been submitted to the NCBI National Center for Biotechnology Information Gene Expression Omnibus (NCBI-GEO) repository and assigned the accession number GSE240807.

## SUPPLEMENTAL MATERIAL

Supplemental figures mbio.02424-23-s0001.docx Fig. S1 to S4.

Tables S1 and S2 mbio.02424-23-s0002.xlsx Differentially expressed genes.

Tables S3, S4, and S5 mbio.02424-23-s0003.pdf Strains, plasmids and cosmids, and oligonucleotides.

Video captions mbio.02424-23-s0004.pdf Captions for Videos S1 and S2

Video S1 mbio.02424-23-s0005.mp4 Wild-type *S. venezuelae* grown on MYMG.

Video S2 mbio.02424-23-s0006.mp4 Wild-type *S. lividans* grown on MYM and MYMG.

ASM does not own the copyrights to Supplemental Material that may be linked to, or accessed through, an article. The authors have granted ASM a non-exclusive, world-wide license to publish the Supplemental Material files. Please contact the corresponding author directly for reuse.
