# Supplementary material for: Redefining development in Streptomyces venezuelae: integrating exploration into the classical sporulating life cycle
Source: mBio. 2024 Mar 12;15(4):e02424-23. doi: 10.1128/mbio.02424-23 (PMC11005364; doi:10.1128/mbio.02424-23)
Supplement: Supplemental figures — Fig. S1 to S4. [file mbio.02424-23-s0001.docx]

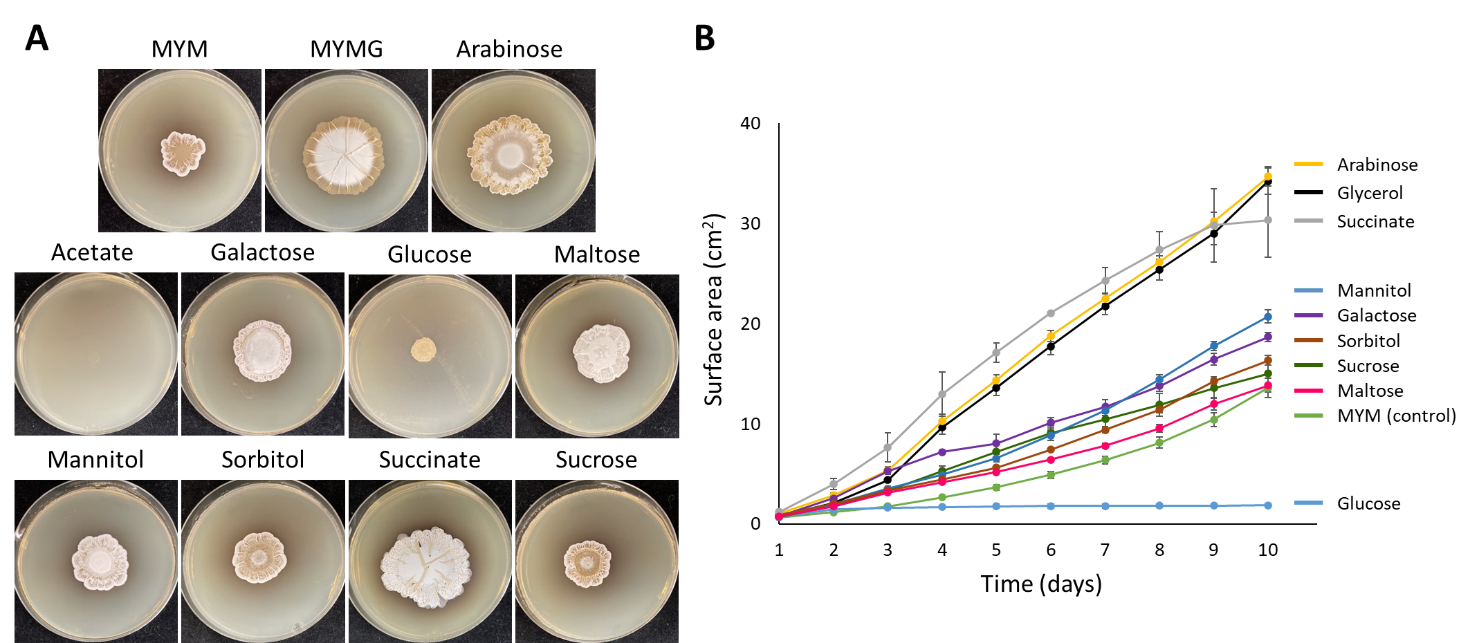


**Supplemental Figure 1 – Effects of other carbon sources on MYM growth. A)** Wild type *S. venezuelae* was spotted to MYM supplemented with different carbon sources at a final concentration of 2% and imaged after 7 days of growth. **B)** Solid growth curves measuring surface area expansion over time for wild type *S. venezuelae* spotted to the media presented in (**A**) with the exception of MYM supplemented with acetate. Error bars represent one standard deviation. *n=*6 for MYM, MYMG and MYM supplemented with arabinose; *n=*3 for all other supplemented cultures.


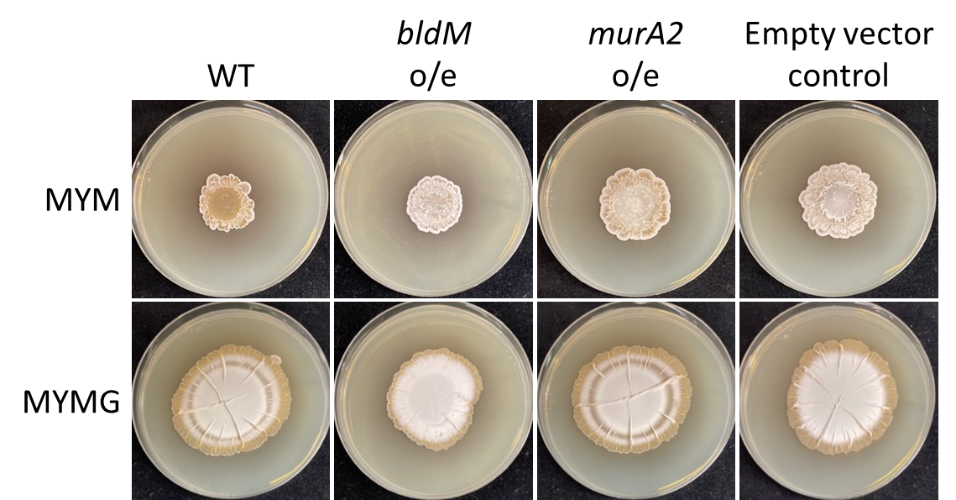


**Supplemental Figure 2 – Overexpressing *bldM* or *murA2* fails to recapitulate a *whiI* overexpression phenotype.** Coding sequences for *bldM* and *murA2* were cloned under the control of the strong constitutive *ermE** promoter, and the resulting constructs were introduced into the wild type and then spotted to different media. A wild type control strain carrying the integrating plasmid with *ermE** alone was included for comparison (right column). Images were taken after 7 days of growth.

**
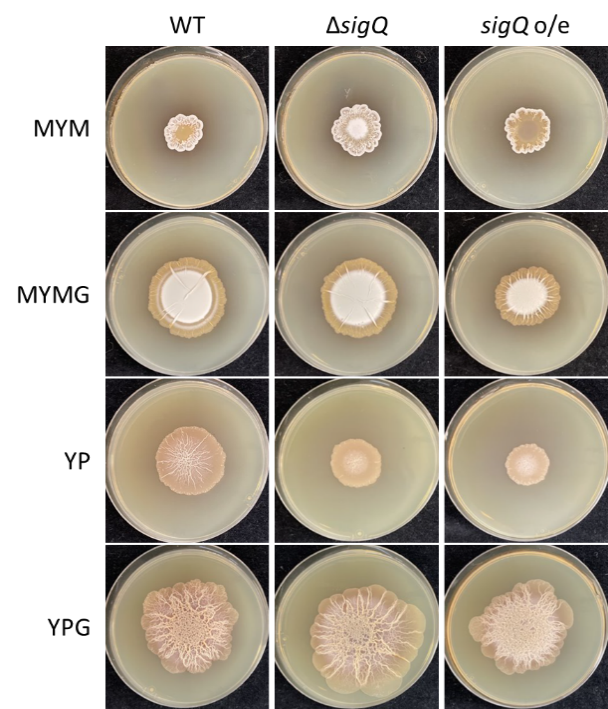
**

**Supplemental Figure 3 – Contribution of *sigQ* to *S. venezuelae* development.** *S. venezuelae* strains carrying a deletion in the *sigQ* or a plasmid with *sigQ* under the strong constitutive promoter *ermE** were spotted to MYM and MYMG and photographed after 7 days of growth.


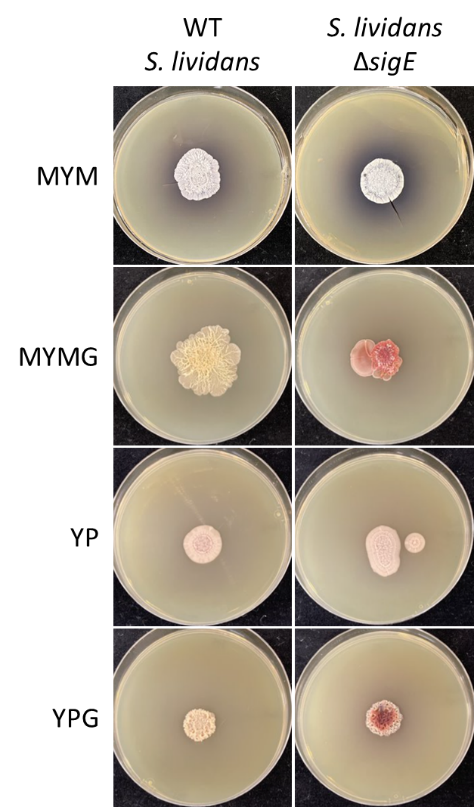


**Supplemental Figure 4 – SigE contributes to MYMG exploration by *S. lividans*.** The homolog of *sigE* was deleted in *S. lividans* and spotted alongside the wild type strain on different media. Photos were taken at 14 days of growth.
