# Supplementary material for: Redefining development in Streptomyces venezuelae: integrating exploration into the classical sporulating life cycle
Source: mBio. 2024 Mar 12;15(4):e02424-23. doi: 10.1128/mbio.02424-23 (PMC11005364; doi:10.1128/mbio.02424-23)
Supplement: Tables S3, S4, and S5 — Strains, plasmids and cosmids, and oligonucleotides. [file mbio.02424-23-s0003.pdf]

Supplemental Table 3 – Strains used in this study

Strains	Genotype/characteristics/use	Reference
Streptomyces		
S. venezuelae NRRL B-65442	Wild type	(1)
S. coelicolor A3(2) M145	Wild type	(2)
S. lividans 1326	Wild type	Gift from the JIC
SV13	*S. venezuelae bldM::aac(3)IV* (*vnz_22005*)	(3)
SV77	*S. venezuelae bldD::aac(3)IV* (*vnz_05285*)	(4)
E331	*S. venezuelae desA-D::aac(3)IV*	(5)
E351	*S. venezuelae gylR-D::aac(3)IV* (glycerol catabolism operon; *vnz_06115-30*)	(6)
E352	*S. venezuelae sigQ::aac(3)IV* (*vnz_22610*)	(6) and this work
E332	*S. venezuelae cmlR::aac(3)IV* (*vnz_04400*)	(7)
E333	*S. venezuelae vnz_34785::hyg* (foroxymithine non-ribosomal peptide synthetase; *fxmE*)	(8)
E334	*S. venezuelae cmlR::aac(3)IV vnz_34785::hyg*	(8)
E335	*S. venezuelae desA-D::aac(3)IV vnz_34785::hyg*	(8)
E353	*S. venezuelae whiG::hyg* (*vnz_26215*)	This work
E354	*S. venezuelae whiH::aac(3)IV* (*vnz_27205*)	This work
E355	*S. venezuelae whiI::aac(3)IV* (*vnz_28820*)	This work
E356	*S. venezuelae whiI::hyg*	This work
E357	*S. venezuelae rsiG::hyg* (*vnz_19430*)	This work
E358	*S. venezuelae rsiG::hyg whiH::aac(3)IV*	This work
E359	*S. venezuelae rsiG::hyg whiI::aac(3)IV*	This work

E360	*S. venezuelae sigE::aac(3)IV* (*vnz_15840*)	This work
E361	*S. venezuelae sigE::aac(3)IV whiI::hyg*	This work
E362	*S. lividans sigE::aac(3)IV* (*sli_3698*)	This work
Escherichia coli		
DH5α	Routine cloning	Invitrogen
BW25113/pIJ790	Introducing mutations in cosmid DNA	(9)
ET12567/pUZ8002	Generation of methylation-free plasmid DNA and conjugation into *Streptomyces*	(9)

Supplementary Table 4 – Plasmids and cosmids used in this study

Cosmid/plasmid	Description	Reference
4O01	*S. venezuelae* cosmid carrying *whiH*	Gift from M. Buttner
StE94	*S. coelicolor* cosmid carrying *sigE*	Gift from M. Buttner
Sv-2_B03	*S. venezuelae* cosmid carrying *whiG*	Gift from M. Buttner
Sv-6-D05	*S. venezuelae* cosmid carrying *whiI*	Gift from M. Buttner
pIJ773	Plasmid carrying the *aac(3)IV-oriT* cassette	(9)
pIJ10700	Plasmid carrying the *hyg-oriT* cassette	(10)
pIJ12551	Plasmid carrying the strong *Streptomyces* promoter *ermE**p	(11)
pMS82	Integrative cloning vector: *hyg*, *oriT*, *int* ΦBT1, *attP* ΦBT1	(12)
pMC302	*ermE**p-*vnz_22610* (*sigQ*) cloned into pMS82	(6)
pMC303	*ermE**p-*vnz_22005* (*bldM*) cloned into pMS82	This work
pMC304	*ermE**p-*vnz_28735* (*murA2*) cloned into pMS82	This work
pMC305	*ermE**p-*vnz_15840* (*sigE*) cloned into pMS82	This work
pMC306	*ermE**p-*vnz_27205* (*whiH*) cloned into pMS82	This work
pMC307	*ermE**p-*vnz_28820* (*whiI*) cloned into pMS82	This work
pCR2.1-TOPO	Cloning vector used for creating *sigE* and *rsiG* deletion strains	Invitrogen
pMC308	4-5 kb sequences upstream and downstream of the *sigE* coding sequence cloned into the pCR2.1-TOPO vector	This work
pMC309	4-5 kb sequences upstream and downstream of the *rsiG* coding sequence cloned into the pCR2.1-TOPO vector separated by a *hyg-oriT* cassette	This work

Supplementary Table 5 – Oligonucleotides used in this study

Name	Sequence (5’ to 3’)	Use
whiG ReD FWD	CGACACACCCGACAGCAGAACGGCTCAAGGCAACGCATG***ATTCCGGGGATCCGTCGACC***	Creation and confirmation of the Δ*whiG* mutation
whiG ReD REV	TGGGCACGGCTCCACTGTACGACCGGCTCGCCCCGGTCA***TGTAGGCTGGAGCTGCTTC***	Creation and confirmation of the Δ*whiG* mutation
whiG Up FWD	CCCGGGTTCGAAGATGTGGC	Confirmation of the Δ*whiG* mutation
whiG In REV	GGGTGATGGCGTACGTCTCG	Confirmation of the Δ*whiG* mutation
whiH ReD FWD	GCCGACAAAGGATGCGTGAGTACCCTTGCGCACACCATG***ATTCCGGGGATCCGTCGACC***	Creation and confirmation of the Δ*whiH* mutation
whiH ReD REV	GCCCCCGACTCCGCACTCCCGGCCACGGCCCGCGGATCA***TGTAGGCTGGAGCTGCTTC***	Creation and confirmation of the Δ*whiH* mutation
whiH In FWD	GGATGCTGGAGCACCTCTCCG	Confirmation of the Δ*whiH* mutation
whiH Down REV	AGGGTACATTCACGCCGCCC	Confirmation of the Δ*whiH* mutation
whiI ReD FWD	CGGCTCCGTCCCGCACCCTTCCCCCCAGGAGGCCTGGTG***ATTCCGGGGATCCGTCGACC***	Creation and confirmation of the Δ*whiI* mutation
whiI ReD REV	CCGTCGACAGGCGCCGGCTTTCCGTCGGCCGGTCCGTCA***TGTAGGCTGGAGCTGCTTC***	Creation and confirmation of the Δ*whiI* mutation
whiI Up FWD	CCTGGTGCGATCTGCTCTGTC	Confirmation of the Δ*whiI* mutation
whiI In REV	CGATCACGTCGCGTACTCCG	Confirmation of the Δ*whiI* mutation
livid *sigE* ReD FWD	CCACCGTCGGAGTACGGGGATCGGAAGGCGGTTGACATG***ATTCCGGGGATCCGTCGACC***	Creation and confirmation of the *S. lividans* Δ*sigE* mutation
livid *sigE* ReD REV	TGGTTCTCCGGGTCCCCCTGGGTGTCCGACCGTCGGTCA***TGTAGGCTGGAGCTGCTTC***	Creation and confirmation of the *S. lividans* Δ*sigE* mutation
livid *sigE* Up FWD	CCGTGACGGACAAATCGCCTC	Confirmation of the *S. lividans* Δ*sigE* mutation
livid *sigE* In REV	GCTCGGTCGGCACCTCCTC	Confirmation of the *S. lividans* Δ*sigE* mutation
livid *sigE* Down REV	CTGCGTGGTTCTCCGGGTC	Confirmation of the *S. lividans* Δ*sigE* mutation
sigE TOPO Left arm FWD HindIII	CATCAT<u>AAGCTT</u>GCTTCGTACGCTTCGCGGAG	Creation of the *S. venezuelae sigE* cosmid equivalent
sigE TOPO Left arm REV SpeI	TACTAC<u>ACTAGT</u>CATGGGCATTCGGACCGTCC	Creation of the *S. venezuelae sigE* cosmid equivalent
sigE TOPO Right arm FWD SpeI	CATCAT<u>ACTAGT</u>CGCCCCGCTGTTACACAACC	Creation of the *S. venezuelae sigE* cosmid equivalent
sigE TOPO Right arm REV XbaI	CATCAT<u>TCTAGA</u>GAGGGCCGCTTTGGTGACG	Creation of the *S. venezuelae sigE* cosmid equivalent
sigE ReD FWD	GAACGAAGCAGGTCACGGGGTTCGGAGGCGGTTCGGATG***ATTCCGGGGATCCGTCGACC***	Creation and confirmation of the *S. venezuelae* Δ*sigE* mutation

sigE ReD REV	TGCTGCCGTCGGCTTCGCCGTCTAGGCCGCGCACCGCTC***TGTAGGCTGGAGCTGCTTC***	Creation and confirmation of the *S. venezuelae* Δ*sigE* mutation
sigE Up FWD	GTGCGTCCACCGACGGCTG	Confirmation of the *S. venezuelae* Δ*sigE* mutation
sigE In FWD	GACGCCTCCGTCGACGACC	Confirmation of the *S. venezuelae* Δ*sigE* mutation
sigE Down REV	CGGCCATGGACAGCCCGAC	Confirmation of the *S. venezuelae* Δ*sigE* mutation
rsiG Gibson Up FWD	CTATGGAAAAACGCCAGCAACGCGGCCTTTTTACGGTTCCCGCTGAGCAAGCTCCTGCTG	Creation and confirmation of the Δ*rsiG* mutation
rsiG Gibson Up REV	AATAGGAACTTCGAACTGCAGGTCGACGGATCCCCGGAATCAGATTCGTCCCCTCGACCG	Creation and confirmation of the Δ*rsiG* mutation
rsiG Gibson HygOriT FWD	CGCCTCCGCACCGGTCGAGGGGACGAATCTGATTCCGGGGATCCGTCGACC	Creation and confirmation of the Δ*rsiG* mutation
rsiG Gibson HygOriT REV	CGTCAGGCGAGCAGGTCGTCGACCTGGGTGTAGGCTGGAGCTGCTTCG	Creation and confirmation of the Δ*rsiG* mutation
rsiG Gibson Down FWD	TCTAGAAAGTATAGGAACTTCGAAGCAGCTCCAGCCTACACCCAGGTCGACGACCTGCTC	Creation and confirmation of the Δ*rsiG* mutation
rsiG Gibson Down REV	ATTAAGTTGGGTAACGCCAGGGTTTTCCCAGTCACGACGTGAGCACGGCCACGATCTGC	Creation and confirmation of the Δ*rsiG* mutation
rsiG Gibson TOPO FWD	GTGGCGCGGCTCGGCGCCGGGCAGATCGTGGCCGTGCTCACGTCGTGACTGGGAAAACCC	Creation and confirmation of the Δ*rsiG* mutation
rsiG Gibson TOPO REV	ACGTAACCCTCCGGTACGGGCAGCAGGAGCTTGCTCAGCGGGAACCGTAAAAAGGCCGCG	Creation and confirmation of the Δ*rsiG* mutation
rsiG Up FWD	GTGCAGGAGTGGCACCATGC	Confirmation of the Δ*rsiG* mutation
rsiG In REV	AGGTCCGACAGCTCCACCTC	Confirmation of the Δ*rsiG* mutation
ermE FWD AvrII	ATATCCTAGGAGCCCGACCCGAGCACGC	Cloning *ermE**p into pMS82
ermE REV HindIII	ATATAAGCTTGATCCTACCAACCGGCACGA	Cloning *ermE**p into pMS82
whiH OE FWD HindIII	CATCATAAGCTTCAAGGGTCACGGAAGCAACGC	Creation of *whiH* overexpression construct
whiH OE REV KpnI	CATCATGGTACCCGCCCAATCTTCGTGGCCC	Creation of *whiH* overexpression construct
whiI OE FWD HindIII	CATCATAAGCTTTCACAGCACGGGTTCGCC	Creation of *whiI* overexpression construct
whiI OE REV KpnI	CATCATGGTACCCCAGCAACGGGATCGGAAGC	Creation of *whiI* overexpression construct
bldM OE FWD HindIII	CATCATAAGCTTGCTACAGCTGCGAGAGCCGCG	Creation of *bldM* overexpression construct
bldM OE REV KpnI	CATCTTGGTACCCTCCATGGTGCCCCGACGCC	Creation of *bldM* overexpression construct
sigE OE FWD NdeI	CGAGTCCATATGAGGAACGAAGCAGGTCACGGG	Creation of *sigE* overexpression construct
sigE OE REV XhoI	CATCATCTCGAGCGTCGGCTTCGCCGTCTAG	Creation of *sigE* overexpression construct

murA2 OE FWD NdeI	CATCATCATATGCCTACCCCAACTGCGAGTG	Creation of *murA2* overexpression construct
murA2 OE REV SpeI	CATCATACTAGTGGAGGCCTGGGACTCGATGAC	Creation of *murA2* overexpression construct

Cassette-specific sequences have been bolded and italicized. Restriction enzyme recognition sequences are underlined.

1. Gomez-Escribano JP, Holmes NA, Schlimpert S, Bibb MJ, Chandra G, Wilkinson B, Buttner MJ, Bibb MJ. 2021. *Streptomyces venezuelae* NRRL B-65442: genome sequence of a model strain used to study morphological differentiation in filamentous actinobacteria . J Ind Microbiol Biotechnol.

2. Kieser T, Bibb MJ, Buttner MJ, Chater KF. 2000. Practical *Streptomyces* Genetics. John Innes Foundation.

3. Al-Bassam MM, Bibb MJ, Bush MJ, Chandra G, Buttner MJ. 2014. Response regulator heterodimer formation controls a key stage in *Streptomyces* development. PLoS Genet 10:e1004554.

4. Tschowri N, Schumacher MA, Schlimpert S, Chinnam NB, Findlay KC, Brennan RG, Buttner MJ. 2014. Tetrameric c-di-GMP mediates effective transcription factor dimerization to control *Streptomyces* development. Cell 158:1136–1147.

5. Jones SE, Pham CA, Zambri MP, McKillip J, Carlson EE, Elliot MA. 2019. *Streptomyces* volatile compounds influence exploration and microbial community dynamics by altering iron availability. mBio 10:e00171-19.

6. Shepherdson EMF, Netzker T, Stoyanov Y, Elliot MA. 2022. Exploratory growth in *Streptomyces venezuelae* involves a unique transcriptional program, enhanced oxidative stress response, and profound acceleration in response to glycerol. J Bacteriol 204:e00623-21.

7. Zhang X, Andres SN, Elliot MA. 2021. Interplay between nucleoid-associated proteins and transcription factors in controlling specialized metabolism in *Streptomyces*. mBio 12:e01077-21.

8. Shepherdson EMF, Elliot MA. 2022. Cryptic specialized metabolites drive *Streptomyces* exploration and provide a competitive advantage during growth with other microbes. Proc Natl Acad Sci 119:e2211052119.

9. Gust B, Challis GL, Fowler K, Kieser T, Chater KF. 2003. PCR-targeted *Streptomyces* gene replacement identifies a protein domain needed for biosynthesis of the sesquiterpene soil odor geosmin. Proc Natl Acad Sci U S A 100:1541–1546.

10. Gust B, Chandra G, Jakimowicz D, Yuqing T, Bruton CJ, Chater KF. 2004. λ Red-mediated genetic manipulation of antibiotic-producing *Streptomyces*. Adv Appl Microbiol 54:107–128.

11. Sherwood EJ, Hesketh AR, Bibb MJ. 2013. Cloning and analysis of the planosporicin lantibiotic biosynthetic gene cluster of *Planomonospora alba*. J Bacteriol 195:2309–21.

12. Gregory MA, Till R, Smith MCM. 2003. Integration site for *Streptomyces* phage ϕBT1 and development of site-specific integrating vectors. J Bacteriol 185:5320–5323.
