# Supplementary material for: Redefining development in Streptomyces venezuelae: integrating exploration into the classical sporulating life cycle
Source: mBio. 2024 Mar 12;15(4):e02424-23. doi: 10.1128/mbio.02424-23 (PMC11005364; doi:10.1128/mbio.02424-23)
Supplement: Video captions — Captions for Videos S1 and S2 [file mbio.02424-23-s0004.pdf]

SUPPLEMENTAL VIDEO CAPTIONS:

Supplemental Video 1: Wild type *S. venezuelae* grown on MYMG, with images captured every hour for 410 hours.

Supplemental Video 2: Wild type *S. lividans* grown on MYM (left) and MYMG (right), with images captured every hour for 410 hours.
